# Ginger Straw Waste-Derived Porous Carbons as Effective Adsorbents toward Methylene Blue

**DOI:** 10.3390/molecules24030469

**Published:** 2019-01-28

**Authors:** Wenlin Zhang, Huihe Li, Jianmin Tang, Hongjia Lu, Yiqing Liu

**Affiliations:** Chongqing Key Laboratory of Economic Plant Biotechnology, Collaborative Innovation Center of Special Plant Industry in Chongqing, Institute of Special Plants, College of Forestry & Life Science, Chongqing University of Arts and Sciences, Yongchuan 402160, China; zhangwenlin88519@126.com (W.Z.); lihuihe@163.com (H.L.); aaluhongjia@163.com (H.L.); liung906@163.com (Y.L.)

**Keywords:** ginger straw, porous carbons, adsorbent, methylene blue

## Abstract

In this work, ginger straw waste-derived porous carbons, with high adsorption capacity, high adsorption rate, and good reusability for removing the toxic dye of methylene blue from wastewater, were prepared by a facile method under oxygen-limiting conditions. This study opens a new approach for the utilization of ginger straw waste, and the porous materials can be employed as great potential adsorbents for treating dye wastewater.

## 1. Introduction

Synthetic dyes are serious pollutants in water due to their extensive use in textile, paper, leather, and printing industries [1]. Due to their potential toxicities for humans and aquatic environment [2], it is very necessary to remove these dyes from wastewater. Methylene blue (MB) is a typical cationic dye which can cause diarrhea, difficult breathing, vomiting, and gastritis in people [3]. To date, many methods, including adsorption, chemical oxidation, and photocatalysis, have been employed to remove MB, and adsorption is the most effective and economical approach [4]. 

Biomass carbon, a carbonized product of biomass created in an oxygen-limiting condition, has many potential applications, such as remediation of contaminated soils, soil fertility improvement, and treatment of industrial wastewater [5,6]. In particular, it is of great interest as an adsorbent for MB removal owing to the pore structure and surface groups. Generally, MB molecules could be trapped by meso/micropores, and macropores are favorable for accelerating the mass transfer process [7,8]. Recently, several carbons derived from various crop wastes, such as wheat straw, rice straw, rice husk, and peanut shell, among others, were developed [9,10,11,12], and their max adsorption capacities (*q*_m_) for MB were 46.6, 62.5, 40.59, 6.76 mg g^−1^, respectively. Among these examples, it was found that the *q*_m_ values of straw-derived carbons were higher. However, for practical application, their *q*_m_ and pore structures still need to be improved. Therefore, it is highly desirable to prepared porous carbons with high adsorption capacity of MB.

**Figure 1:**
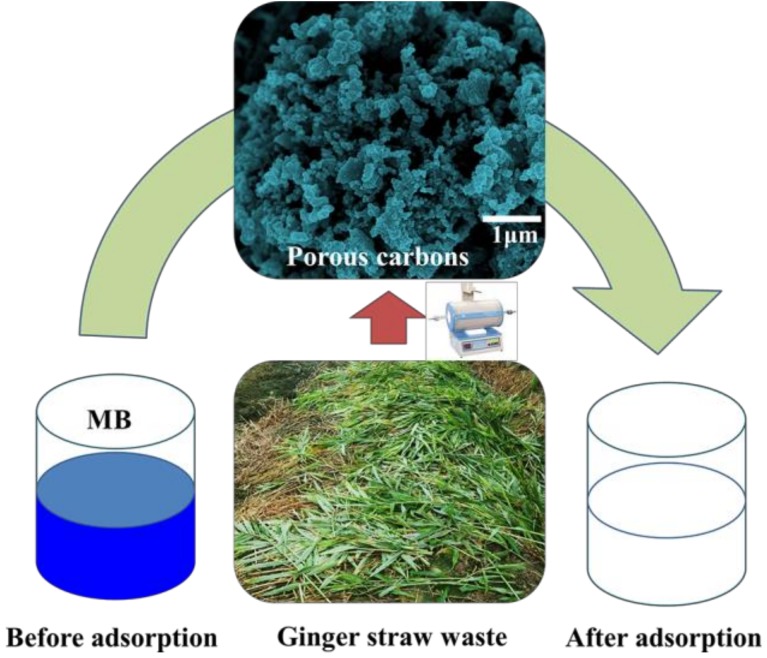
Illustration of the procedure for synthesis of ginger straw-derived porous carbon (GSPC) and its application in methylene blue (MB) removal.

Ginger is one of the most ancient plant spices in the world, and has been widely employed as an additive for various food, beverages, and medicine [13]. Ginger straw (GS) is a byproduct of ginger farming. Noticeably, a huge amount of ginger straw is commonly discarded as waste in the field, which causes many environment problems. For waste utilization, using GS as a precursor to prepare carbons is considered. To our knowledge, the use of unique porous carbons derived from GS as adsorbents for MB has not been reported. In this work, GS-derived porous carbons (GSPCs) were prepared, showing high adsorption capacity and good reusability for MB removal (Figure 1), thus being potential adsorbents for treating MB wastewater.

## 2. Results and Discussion

**Figure 2:**
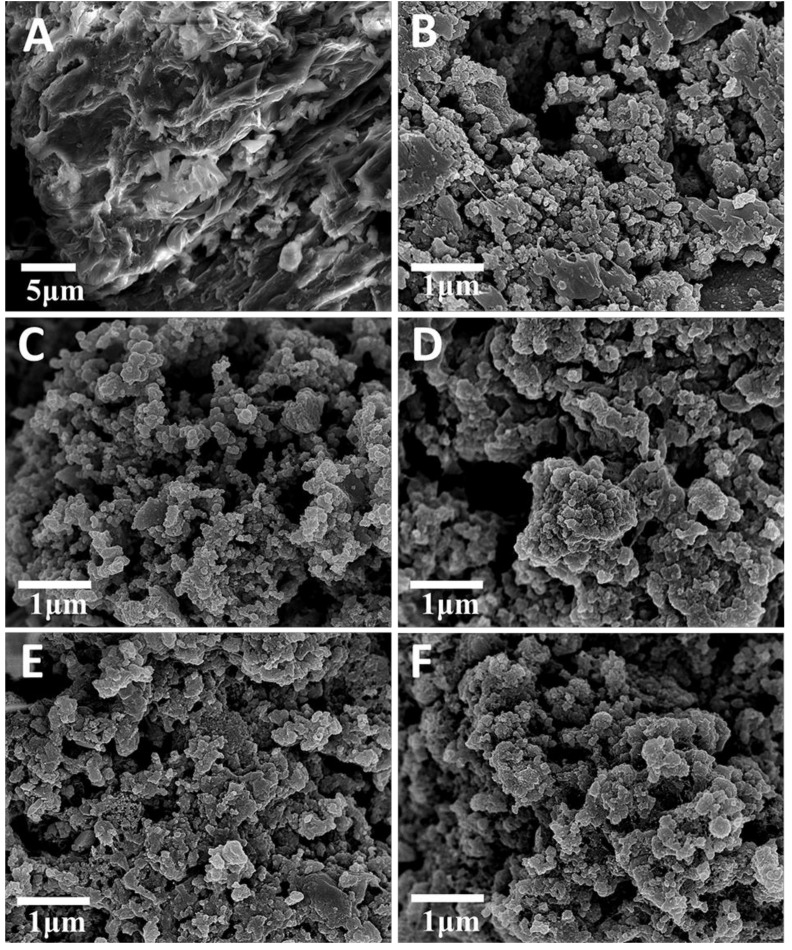
SEM images of (**A**) GS and GSPC prepared at (**B**) 300, (**C**) 400, (**D**) 500, (**E**) 600, and (**F**) 700 °C.

**Figure 3:**
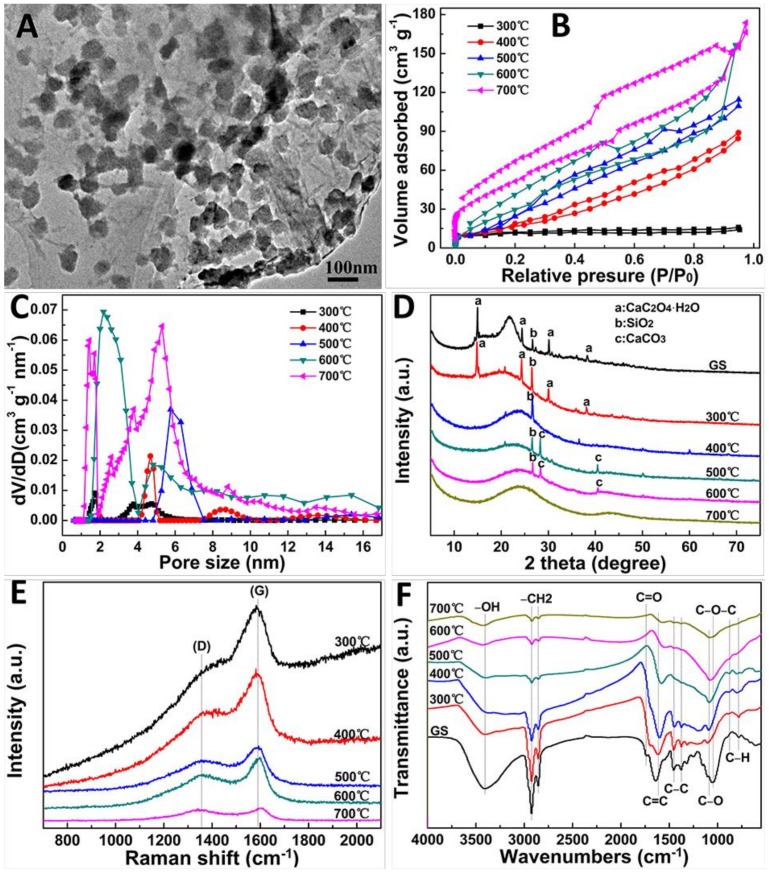
(**A**) TEM image of GSPC prepared at 700 °C. (**B**) Nitrogen adsorption–desorption isotherms, (**C**) pore size distributions, (**D**) XRD patterns, (**E**) Raman, and (**F**) FTIR spectra of GSPC prepared at 300, 400, 500, 600, and 700 °C.

Figure 2A showed the scanning electron microscope (SEM) image of GS, and its structures appeared obviously changed after heat treatment using various temperatures (Figure 2B–F). However, no significant differences were found in the morphology of GSPC prepared at 300–700 °C. Moreover, the transmission electron microscope (TEM) image (Figure 3A) revealed that some nanoparticles (dark portions) derived from the mineral phases existed in GSPC, which were consistent with the previously reported biomass carbons [14,15]. 

The nitrogen adsorption–desorption isotherms (Figure 3B, Table S1) indicated that the specific surface area (SSA) of GSPC increased from 50.5 to 171.5 m^2^g^−1^ with the increase of temperature from 300 to 700 °C, respectively, which were much higher than some other reported biomass carbons, as in Table S2. Meanwhile, the isotherms displayed a type-I sorption isotherm with steep nitrogen uptakes at P/P0 < 0.05 (Figure 3B), indicating that GSPC had a large amount of micropores [16]. Furthermore, the pore size distributions revealed that the pore sizes of GSPC were mainly located at micropore and mesopore regions (Figure 3C, Table S1). In addition, the pore size distributions were changed drastically on various heating temperatures. The distributions seemed to be monomodal at 500 °C, bimodal at 400 and 600 °C, and trimodal at 300 and 700 °C, showed a typical shape of “V”. This result suggested that the heating temperature significantly affected the pore size distributions of GSPC. It was believed that parts of pores were destroyed or involved into larger ones when the temperatures increased from 300 to 500 °C, while the some new mesopores or micropores were produced when the temperatures increased to 600 and further to 700 °C. Such pore change might have a deep relation to the heating temperature, specific surface area, pore volume and surface groups of GSPC. For the detail mechanism for formation of pores, we thought that a dehydration of GS occurred during the carbonization process, which resulted in the charring and aromatization of carbon skeleton, and so created the porous structures [17]. According to the above analysis, GSPC indeed had abundant meso/micropores. It should be emphasized that these formed pores were favorable for entrapping MB molecules, and MB molecules can diffuse into the surface of GSPC rapidly [7]. To analyze the phase composition of GSPC, X-ray diffraction (XRD) was performed, as shown in Figure 3D. The major crystalline phases in the GS and GSPC prepared at 300 °C were whewellite (CaC_2_O_4_·H_2_O) and quartz (SiO_2_) [5,18]. As the temperature increased, the whewellite decomposed at 400 °C and transformed into calcite (CaCO_3_) at 500 and 600 °C. Calcite was degraded during N_2_ condition (CaCO_3_ → CaO + CO_2_) as the temperature increased to 700 °C, and the XRD signal of calcite disappeared completely, which agreed with the reported biomass carbons [5,18]. The broad peaks at about 24° corresponded to (002) reflections of the disordered carbon layer [5]. Raman spectra revealed that the two peaks at 1388 cm^−1^ (D-band) and 1583 cm^−1^ (G-band) were associated with sp3 and sp2 hybridized carbons [10] in GSPC prepared at 300–700 °C (Figure 3E), respectively. Fourier transform-infrared (FTIR) spectra of GSPC in Figure 3F showed that the bands at 3404, 2923, 2855, 1740, 1612, 1452, 1376, 1045 cm^−1^ corresponded to –OH, –CH_2_, C=O, C=C, C–C, C–O–C, respectively [19,20]. FTIR results suggested that the abundant oxygenated groups, such as –COOH and –OH, existed in GSPC, which likely played important roles in the MB adsorption process owing to electrostatic interactions [21,22].

**Figure 4:**
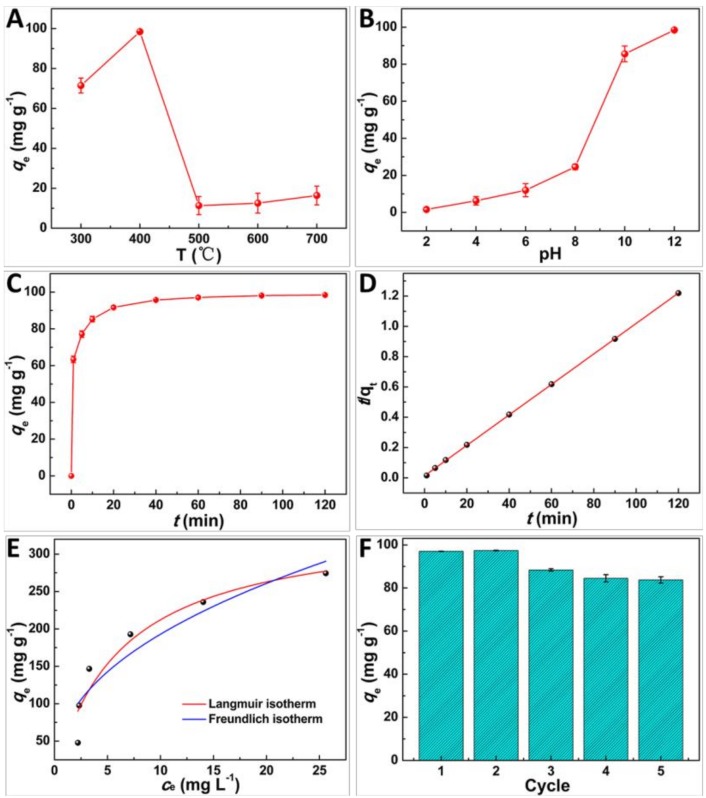
Effect of (**A**) heat treatment temperature (*c*_0_: 100 mg L^−1^, 2 h, 25 °C, pH: 12), (**B**) pH (*c*_0_: 100 mg L^−1^, 2 h, 25 °C), (**C**) contact time (*c*_0_: 100 mg L^−1^,pH: 12, 25 °C), (**D**) pseudo-second order kinetics; (**E**) Langmuir and Freundlich isotherms of MB adsorption on GSPC; (**F**) the reusability of GSPC toward MB adsorption (*c*_0_: 100 mg L^−1^, pH: 12).

Interestingly, GSPC obtained at 400 °C exhibited a higher adsorption capacity (*q*_e_) of MB than those of the materials prepared at 300, 500, 600, and 700 °C (Figure 4A), and was chosen as the potential adsorbent. Fundamentally, pH is an important parameter for MB adsorption [20]. Clearly, as pH increased from 2 to 12, *q*_e_ improved significantly from 1.6 to 98.5 mg g^−1^ (Figure 4B). MB is a positively charged molecule [23], and GSPC is negatively charged due to the surface group of –COOH (Figure 3F). With pH increase, the enhanced electrostatic interaction between MB and GSPC led to a higher *q*_e_ [22]. On the contrary, the decreased *q*_e_ at lower pH was due to the competition between protons and MB molecules for the adsorption sites of GSPC. Moreover, *q*_e_ increased with the raised MB initial concentration (*c*_0_) (Figure S1A), revealing the favorable adsorption at higher *c*_0_, which gave a larger driving force to overcome mass transfer resistance of MB from aqueous phase to solid phase [24]. It can be seen that the adsorption rate was quite high (63.4 mg g^−1^ min^−1^, Figure 4C) in the first minute when MB and GSPC were not fully mingled. Obviously, the electrostatic interaction played an important role in the adsorption progress along with mosaiced adsorption. Then, the rate decreased because of the repulsive forces between the free MB molecules in aqueous phase and the adsorbed MB molecules on GSPC [4]. Ultimately, the adsorption reached an equilibrium at 60 min. To explore the kinetics of MB adsorption on GSPC, pseudo-first order (PFO) and pseudo-second order (PSO) models were employed (Figure S1B, 4D). Clearly, the correlation coefficients (*R*^2^) of PSO (0.9999) (Table S3) were higher than that of PFO (0.9714), while the calculated *q*_e,cal_ from PSO was in good agreement with the experimental *q*_e,exp_, indicating PSO was more suitable to describe the adsorption process [25]. Langmuir (L) and Freundlich (F) isotherm models were used to depict the solute–surface interaction between GSPC and MB, as well as to quantitatively analyze the *q*_m_. As in Figure 4E and Table S4, *R*^2^ from L (0.9078) was higher than that of F (0.8412), suggesting L was more suitable for evaluating the adsorption behavior [25]. This indicated that MB adsorption was a monolayer adsorption on the heterogeneous surface of GSPC, which agreed with other reported biomass carbons [26,27]. On the basis of L equation, the *q*_m_ of MB on GSPC was 345.0 mg g^−1^ at 25 °C, which was higher than those of other reported biomass carbons as in Table S5, indicating the promising application of GSPC for MB removal. The reusability of the adsorbents is vital for practical application. As in Figure 4F, the *q*_e_ decreased a little after five cycles from 97.0 to 83.7 mg g^−1^. Fundamentally, some dye molecules were adsorbed in the mesopores [8], whereas the desorption did not remove the adsorbed dye completely, resulting in a slight decrease of *q*_e_ for next cycle. However, SEM image in Figure S2 depicted that the morphology of GSPC was also maintained even after five cycles. Moreover, the elemental compositions of GSPC before the first cycle and after the fifth cycle showed no obvious changes (Figure S3). Together with the previous reports [4,7,8,28], we found the GSPC to have good stability and reusability for MB removal.

A potential mechanism for the high adsorption capacity and adsorption rate of MB on GSPC was proposed. Fundamentally, adsorption is a physicochemical process involving the mass transfer of a solute from liquid phase to adsorbent surface and the interactions between dye molecules and adsorbent [4,8]. The high SSA and abundant meso/micropores of GSPC could benefit from improving adsorption capacity, of which SSA provided rich adsorption sites and abundant mesopores could trap more MB molecules [8]. On the basis of Table S1, GSPC prepared at ≥500 °C possessed larger SSA and pore volume than those prepared at ≤400 °C, but their adsorption capacities was lower (Figure 4A). However, the number of surface groups, such as –COOH and –OH (Figure 3F) on GSPC prepared at ≤400 °C was higher than those of the higher temperature, indicating that interactions such as electrostatic interactions, H-bonding interactions, and π–π stacking between MB molecules and GSPC was mainly responsible for the adsorption behavior [29], and played critical roles in the adsorption capacity and adsorption rate of GSPC. Therefore, the adsorption enhancement mechanism of GSPC could be mainly attributed to strong interactions between MB molecules and GSPC, as well as its porous structures.

## 3. Material and Methods

The material and methods were provided in the Supplementary Materials.

## 4. Conclusions

In this work, ginger straw-derived porous carbons were synthesized successfully via a facile method. The obtained materials had high adsorption ability toward MB in terms of high adsorption capacity, high adsorption rate, and good reusability. The kinetics and isotherm data were well depicted by a pseudo-second order kinetics model and Langmuir model, respectively. The adsorption enhancement mechanism of GSPC was mainly due to interactions between MB molecules and GSPC, as well as its porous structures. Our study provided a new way for the utilization of ginger straw wastes, and the as-prepared materials hold a great potential for MB removal from wastewater.

## Supplementary Materials

The Supplementary Materials are available online at http://www.mdpi.com/1420-3049/24/3/469/s1. Figure S1. Effect of (A) *c*_0_ (2 h, 25 °C, pH: 12) for MB adsorption on GSPC. (B) Pseudo-second order kinetic model of MB adsorption on GSPC; Figure S2. SEM image of GSPC after five cycles of adsorption–desorption; Figure S3. EDS spectra of GSPC (A) before first cycle and (B) after fifth cycle; Table S1. Specific surface area and pore characteristics of GSPC at various temperatures; Table S2. Comparison of the specific surface area of other biomass carbons; Table S3. Kinetic model parameters of MB adsorption on GSPC; Table S4. Isotherm model parameters of MB adsorption on GSPC; Table S5. Comparison of the max adsorption capacities of MB on various biomass carbons.
