# Supplementary material for: Ginger Straw Waste-Derived Porous Carbons as Effective Adsorbents toward Methylene Blue
Source: Molecules. 2019 Jan 28;24(3):469. doi: 10.3390/molecules24030469 (PMC6384592; doi:10.3390/molecules24030469)
Supplement: Supplementary file 1 [file molecules-24-00469-s001.pdf]

Supplementary Material

Ginger straw waste derived porous carbons as effective adsorbents toward methylene blue

Wenlin Zhang[1], Huihe Li[1], Jianmin tang[1*], Hongjia Lu[1], and Yiqing Liu[1]

[1]*Chongqing Key Laboratory of Economic Plant Biotechnology, Collaborative Innovation Center of Special Plant Industry in Chongqing, Institute of Special Plants, College of Forestry & Life Science, Chongqing University of Arts and Sciences, Yongchuan 402160, China*

* Correspondence: tangjmjy@163.com

Supplementary caption:

Figure S1. Effect of (A) c_0 (2 h, 25°C, pH: 12) for MB adsorption on GSPC. (B) Pseudo-second -order kinetic model of MB adsorption on GSPC.

Figure S2. SEM image of GSPC after five cycles of adsorption-desorption.

Figure S3. EDS spectra of GSPC (A) before first cycle and (B) after fifth cycle.

Table S1. Specific surface area and pore characteristics of GSPC at various temperatures.

Table S2. Comparison of the specific surface area of other biomass carbons.

Table S3. Kinetic model parameters of MB adsorption on GSPC.

Table S4. Isotherm model parameters of MB adsorption on GSPC.

Table S5. Comparison of the max adsorption capacities of MB on various biomass carbons.

Materials and methods

Materials

Ginger straw was collected from the ginger field. Methylene blue (MB) were purchased from Sigma-Aldrich Co. LLC. (St Louis, USA) and other chemicals were all obtained from Chengdu Kelong Chemical Reagent Co. (Sichuan, China). Ultrapure water was used for all experiments.

Characterizations

The morphology of prepared samples was obtained on a Hitachi SU8220 field-emission scanning electron microscope (SEM, Japan) operated at 20 kV. X-ray diffraction (XRD) patterns were performed by a Bruker D8 diffractometer (Bruker, Germany) using the CuKa radiation. Fourier transform-infrared (FTIR) spectra were recorded using a Nicolet 6700 spectrophotometer (Thermo Fisher, USA). Nitrogen adsorption-desorption isotherm was measured using a Quadrasorb instrument (Quantachrome, USA) at 150°C, and data analysis was performed with Quantachrome software. The average pore sizes were calculated from the nitrogen adsorption isotherms according to the nonlocal density functional theory (NLDFT) model. UV-vis absorption spectra were performed on a Perkin Elmer Lambda 25 spectrophotometer.

Synthesis of ginger straw derived porous carbons (GSPC)

In a typical process, 5.0 g ginger straw powder was transferred in a tube furnace under nitrogen flow with a heating rate of 5ºC min^{-1} and then held at 300, 400, 500, 600, 700 ºC for 1 h, respectively. Then, the as-prepared samples were washed with ultrapure water. Finally, the

product was dried at 60 ºC in an oven for 24 h.

Batch adsorption experiments

Batch adsorption experiments were performed in glass bottles containing 10 mg of GSPC and 10 mL of MB aqueous solutions with the initial concentrations ranging from 100 and 300 mg L^{-1}. Subsequently, the mixtures were shaken in a shaking incubator at different times with a shaking speed of 200 rpm. Then, the mixtures were centrifuged and the supernatant concentrations of MB were determined by a UV-Vis spectrophotometer at 664 nm. The effect of solution pH on MB adsorption on GSPC was investigated by varying pH value from 2 to 12. The effect of temperature on MB removal was also studied by keeping the temperature at 25-55ºC. The adsorption capacity of MB by GSPC was calculated via the following equations:

$$q_{\mathrm{e}} = \frac{(c_0 - c_{\mathrm{e}})V}{m} \quad (1)$$

q_e (mg g^{-1}) represents the equilibrium adsorption capacity of MB on GSPC, c_0 (mg L^{-1}) and c_e (mg L^{-1}) are the initial and equilibrium concentrations of MB, respectively, V (L) is the volume of solution, and m (g) is the mass of GSPC.

Adsorption kinetic and isotherm models

To understand the adsorption dynamics of MB-GSPC system in relation to time and depict the nature of solute-surface interaction between adsorbent and MB as well as to investigate the performance of adsorbent, three kinetic models (pseudo-first-order and pseudo-second-order) and two isotherm models (Langmuir and Freundlich) were studied. The equations can be listed as follows:

$$\log(q_{\mathrm{e}} - q_{\mathrm{t}}) = \log q_{\mathrm{e}} - \frac{k_1 t}{2.303} \quad (2)$$

$$\frac{t}{q_{\mathrm{t}}} = \frac{1}{k_2 q_{\mathrm{e}}^2} + \frac{t}{q_{\mathrm{e}}} \quad (3)$$

$$q_{\mathrm{e}} = \frac{b q_m C_{\mathrm{e}}}{1 + b C_{\mathrm{e}}} \quad (4)$$

$$q_{\mathrm{e}} = k C_{\mathrm{e}}^{1/n} \quad (5)$$

q_t (mg g^{-1}) represents adsorption capacity of MB on GSPC at any time t (min), k_1(min^{-1}) and k_2 (g mg^{-1} min^{-1}) are pseudo-first-order and pseudo-second-order adsorption rate constants, respectively, t is contact time (min). k_i (mg g^{-1} $h^{-1/2}$) is intraparticle diffusion rate constant and c_i (mg g^{-1}) is a constant. q_m (mg g^{-1}) is maximum adsorption capacity of MB on GSPC, b (L mg^{-1}) is Langmuir adsorption constant, k is indicator of adsorption capacity, and $1/n$ represents heterogeneity factor.

Regeneration experiments

For the regeneration study, 10 mg GSPC were added to 10 mL MB solution (100 mg L^{-1}) at pH 12 and the mixture was shaken at 200 rpm for 60 min at 25 ºC. After adsorption and centrifugation, the supernatant MB solution was discarded leaving GSPC. Then, the MB-adsorbed GSPC were added to 10 mL of absolute ethanol and shaken at 200 rpm for 10 min. Subsequently, the GSPC were isolated from the solution by centrifugation and used for

next cycle. The final concentrations of MB were determined by UV-vis spectra. The adsorption-desorption processes as described were carried out successively for five times.

Table S1. Specific surface area and pore characteristics of GSPC at various temperatures.

T(ºC)	SSA (m^2g^{-1})	Pore Volume (cm^3g^{-1})	Average Pore Size (nm)
300	50.50	0.019	1.70
400	80.60	0.037	4.68
500	89.04	0.039	5.76
600	134.45	0.230	2.18
700	171.50	0.245	5.30

Table S2. Comparison of the specific surface area of other biomass carbons.

Biomass carbons	S_{BET} (m^2g^{-1})	References
Wheat straw derived carbons	0.75	[1]
Rice straw derived carbons	36.7	[2]
Corn straw derived carbons	49.4	[3]
Rice husk derived carbons	5.24	[1]
Walnut shell derived carbons	1.56	[1]
Peach branch derived carbons	3.88	[1]
Sewage sludge derived carbons	25	[4]
Eucalyptus saw dust derived carbons	1.57	[5]
**Ginger straw derived porous carbons**	**171.5**	**This work**

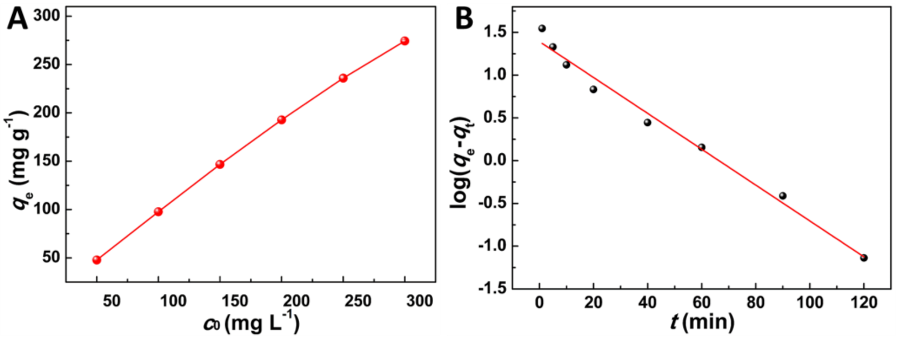

Figure S1. Effect of (A) c_0 (2 h, 25°C, pH: 12) for MB adsorption on GSPC. (B) Pseudo-second -order kinetic model of MB adsorption on GSPC.

Table S3. Kinetic model parameters of MB adsorption on GSPC.

		Pseudo-first-order kinetics			Pseudo-second-order kinetics		
c_0 (mg L^{-1})	$q_{e,exp}$ (mg g^{-1})	$q_{e,cal}$ (mg g^{-1})	k_1 (min^{-1})	R^2	$q_{e,cal}$ (mg g^{-1})	k_2 (g mg^{-1} min^{-1})	R^2
100	98.4	24.7	0.05	0.9714	99.4	0.0074	0.9999

Table S4. Isotherm model parameters of MB adsorption on GSPC.

Temperature (°C)	Langmuir			Freundlich		
	q_m (mg g^{-1})	b (L mg^{-1})	R^2	k	$1/n$	R^2
25	345.0	0.16	0.9078	70.81	2.30	0.8412

Table S5. Comparison of the max adsorption capacities of MB on various biomass carbons.

Biomass carbons	q_m (mg g^{-1})	References
wheat straw derived carbons	46.6	[6]
Rice straw derived carbons	62.5	[7]
Rice husk derived carbons	40.59	[8]
Weeds derived carbons	39.68	[9]
Bamboo derived carbons	4.78	[10]
Peanut hull derived carbons	68.03	[11]
Palm bark derived carbons	2.66	[12]
Hickory wood derived carbons	16.3	[13]
Anaerobic digestion residue derived carbons	9.50	[14]
**Ginger straw derived porous carbons**	**345.0**	**This work**

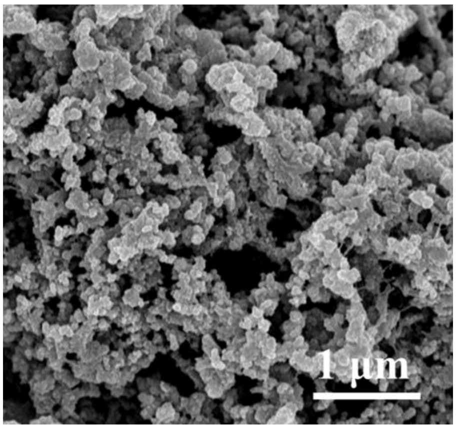

Figure S2. SEM image of GSPC after five cycles of adsorption-desorption.

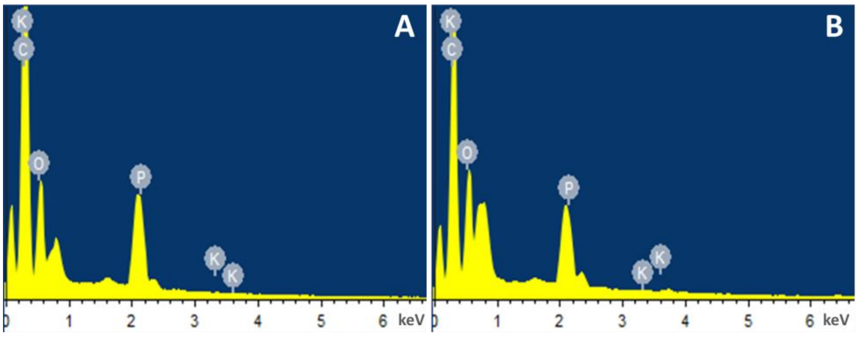

Figure S3. EDS spectra of GSPC (A) before first cycle and (B) after fifth cycle.

References

[1] Wang, Y.; Liu, R. Comparison of characteristics of twenty-one types of biochar and their ability to remove multi-heavy metals and methylene blue in solution. *Fuel Process. Technol.* **2017**, *160*, 55-63.

[2] Lu, K.; Yang, X.; Gielen, G.; Bolan, N.; Ok, Y.S.; Niazi, N.K.; Xu, S.; Yuan, G.; Chen, X.; Zhang, X.; Liu, D.; Song, Z.; Liu, X.; Wang, H. Effect of bamboo and rice straw biochars on the mobility and redistribution of heavy metals (Cd, Cu, Pb and Zn) in contaminated soil. *J. Environ. Manage.* **2017**, *186*, 285-292.

[3] Sun, J.; Lian, F.; Liu, Z.; Zhu, L.; Song, Z. Biochars derived from various crop straws: characterization and Cd (II) removal potential. *Ecotox. Environ. Safe.* **2014**, *106*, 226-231.

[4] Fan, S.; Wang, Y.; Wang, Z.; Tang, J.; Tang, J.; Li, X. Removal of methylene blue from aqueous solution by sewage sludge-derived biochar: Adsorption kinetics, equilibrium, thermodynamics and mechanism. *J. Environ. Chem. Eng.* **2017**, *5*, 601-611.

[5] Sun, L.; Chen, D.; Wan, S.; Yu, Z. Performance, kinetics, and equilibrium of methylene blue adsorption on biochar derived from eucalyptus saw dust modified with citric, tartaric, and acetic acids. *Bioresour Technol.*, **2015**, *198*, 300-308.

[6] Liu, Y.; Zhao, X.; Li, J.; Ma, D.; Han, R. Characterization of bio-char from pyrolysis of wheat straw and its evaluation on methylene blue adsorption. *Desalin. Water Treat.* **2012**, *46*, 115-123.

[7] Mashhadi, S.; Javadian, H.; Ghasemi, M.; Saleh, T.A.; Gupta, V.K. Microwave-induced H_2SO_4 activation of activated carbon derived from rice agricultural wastes for sorption of methylene blue from aqueous solution. *Desalin. Water Treat.* **2016**, *57*, 21091-21104.

[8] Vadivelan, V.; Kumar, K.V. Equilibrium, kinetics, mechanism, and process design for the sorption of methylene blue onto rice husk. *J. Colloid Interface Sci.* **2005**, *286*, 90-100.

[9] Güzel, F.; Sayğılı, H.; Sayğılı, G.A.; Koyuncu, F.; Yılmaz, C. Optimal oxidation with nitric acid of biochar derived from pyrolysis of weeds and its application in removal of hazardous dye methylene blue from aqueous solution. *J. Clean Prod.* **2017**, *144*, 260-265.

[10] Huff, M. D.; Kumar, S.; Lee, J. W. Comparative analysis of pinewood, peanut shell, and bamboo biomass derived biochars produced via hydrothermal conversion and pyrolysis. *J. environ. Manage.* **2014**, *146*, 303-308.

[11] Gong, R.; Li, M.; Yang, C.; Sun, Y.; Chen, J. Removal of cationic dyes from aqueous solution by adsorption on peanut hull. *J. Hazard. Mater.* **2005**, *121*, 247-250.

[12] Tan, X.; Liu, Y.; Zeng, G.; Wang, X.; Hu, X.; Gu, Y.; Yang, Z. Application of biochar for the removal of pollutants from aqueous solutions. *Chemosphere* **2015**, *125*, 70-85.

[13] Ding, Z.; Wan, Y.; Hu, X.; Wang, S.; Zimmerman, A.R.; Gao, B. Sorption of lead and methylene blue onto hickory biochars from different pyrolysis temperatures: importance of physicochemical properties. *J. Ind. Eng. Chem.* **2016**, *37*, 261-267.

[14] Sun, L.; Wan, S.; Luo, W. Biochars prepared from anaerobic digestion residue, palm bark, and eucalyptus for adsorption of cationic methylene blue dye: characterization, equilibrium, and kinetic studies. *Bioresour Technol.* **2013**, *140*, 406-413.
